# A Characterizing Method of Carbon Nanotubes in Powder Form with Different Packing Densities

**DOI:** 10.3390/mi16060662

**Published:** 2025-05-31

**Authors:** Ruiliang Li, Chuang Yang, Yunlong Zhang, Jian Wang

**Affiliations:** 1 Qingdao Institute for Marine Technology of Tianjin University, Qingdao 266200, China; tjliruiliang@163.com; 2 School of Information and Communication Engineering, Beijing University of Posts and Telecommunications, Beijing 100876, China; chuangyang@bupt.edu.cn; 3 Qingdao ZITN Micro-Electronics Company Limited, Qingdao 266114, China; zhangyunlong@qdzitn.com; 4 School of Microelectronics, Tianjin University, Tianjin 300072, China

**Keywords:** carbon nanotube powders, microwave, density, transmission/reflection, de-embed

## Abstract

A method for characterizing carbon nanotubes (CNTs) in powder form with different packing densities in the microwave regions is proposed. The CNTs were sandwiched between two dielectric walls in (Polyvinyl Chloride) PVC and put in a waveguide shim. We measured the transmission/reflection S-parameters of the waveguide using a Vector Network Analysis (VNA), and the impacts of the PVCs on the measured S-parameters were de-embedded by microwave network analysis. Then, the well-known Nicolson–Ross–Weir (NRW) method was processed to determine the complex permittivity and permeability of the CNTs. Furthermore, we pressed the PVC to increase the packing densities of the CNTs. The results of the characterization can be employed to design microwave devices using the CNTs.

## 1. Introduction

Since carbon nanotubes (CNTs) were discovered by S. Iijima in 1991 [1], the RF/microwave applications of CNTs have attracted strong interest [2]. Knowledge of the complex permittivity and permeability of CNTs in the frequency bands is necessary to implement novel RF/microwave CNT-based components, such as electromagnetic interference shielding packages, interconnection lines, field-effect transistors, etc. [3,4]. Over the past 30 years, carbon nanotubes and their related materials, such as graphene, have played a major role in microwave circuit devices.

Nevertheless, the characterization of CNTs is challenging because it usually requires special preparation of either a thin film [5] or embedding CNTs into a host medium [6] that decreases the accuracy of the extracted parameters [7,8]. Decrossas et al. [9,10] solved the challenge and characterized CNTs and CNT-based materials in powder form with different packing densities over a broadband of frequencies. However, only the complex permittivity of the powders is determined by the method since only the reflection parameter is employed. It is necessary to determine complex permittivity and permeability simultaneously by transmission/reflection methods, because the value of the real part of the relative complex permeability of CNTs may not be one or even negative. The negative values are usually important for the design of the metamaterial [11].

This paper proposes a method to determine the complex permittivity and permeability of CNTs in powder form with different packing densities in the microwave region. As the intrinsic characterization of powder-form materials cannot be determined by traditional transmission/reflection methods, where the powder is mixed with polyester material to set it solid, we proposed a multi-layer process. Furthermore, it is the first time that the packing density of CNTs is controlled by pressing in the transmission/reflection measurement. We developed a uniquely engineered waveguide fixture utilizing PVC sheets with calibrated friction to systematically compress and fix CNT powders within the test region. This allowed us to reproducibly adjust the packing density across a broad range (0.1549–0.4856 g/cm³), which is a significant advancement over previous works that relied on fixed-density or slurry-based methods. The effects of frequency and the packing density on complex permittivity of CNTs are characterized experimentally. There are several advantages of the method used in this study: no special preparation is necessary; complex permittivity and permeability are determined simultaneously; Material Under Test (MUT) can be powdered, granular, and nondestructive. The discussed method equally applies to microwave waveguide and coaxial measurements, though the example discussed herein is performed in the X-band waveguide.

## 2. Methodology

### 2.1. Experimental Procedure

**Figure 1:**
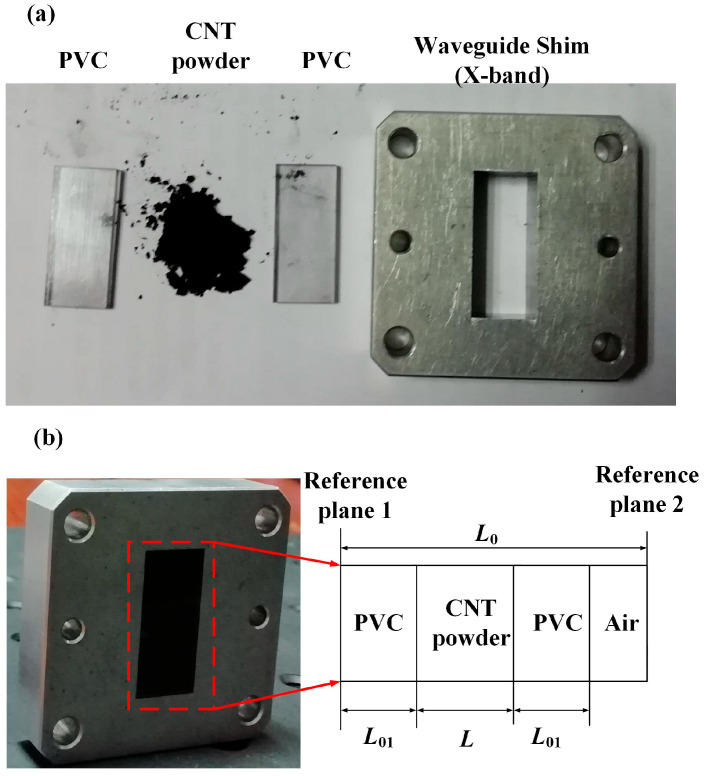
(**a**) Image of the CNTs in powder form and the sample holder before assembling; (**b**) image and schematic of the test setup: the waveguide containing the sample and the two PVC sheets used for performing the measurements in the X-band.

To achieve the expected measurement, we designed an experimental system, as shown in Figure 1a. It shows the image of the CNTs (MWCNTs, >95wt%, inner diameter: 3–5 nm, outer diameter: 8–15 nm, and length: 3–12 μm) in powder form and the sample holder before assembling. Figure 1b shows the image and schematic of the waveguide used to perform the measurements in the X-band (22.86 × 10.16 mm) that has been used in the previous work [12]. As shown in Figure 1b, the waveguide is segmented into four parts: the containment area of the material sample, the air-filled section, and the two (Polyvinyl Chloride) PVC sheets. The containment area is delimited by two dielectric walls in PVC. As the PVC is transparent, we can see that the waveguide section between the PVC is filled with CNT powder. The length of the waveguide is 9.78 mm, which is a 1/4 λ wavelength in the X-band calibration kits, and the length of the two PVC sheets is 1.95 mm. The relative complex permittivity and the relative permeability of the PVC are known and expressed as $\varepsilon_{1r}$ and $\mu_{1r}$.

The thickness of the PVC is 1 mm, and the length and width of the PVC agree well with the internal dimensions of the waveguide. The agreement between the PVC and waveguide, and the frictional force between them is used to compress the CNT. The thickness of the CNT is calculated in Figure 1. The *L*_0_ is 9.78 mm, the L_01_ is 1 mm, and the depth of air in the waveguide is measured by the vernier caliper (resulution: 0.01 mm). Then, the thickness of CNT powder is calculated.

The weight of the CNTs used in this work is approximately 0.15 g, and the volume of the CNTs changes with the press in the PVC sheet. To change the density of the CNTs, we slowly press the PVC sheet, which changes the value of the *L*, which is the thickness of the CNTs. The density of the CNTs changes with the L as shown in Table 1.

Packing density is computed using the following relation

(1) $$\rho =\frac{M}{V}(\frac{g}{cm^{3}})$$

where *M* is the mass of the CNTs in grams weights, and *V* is the volume of the CNTs in cm^3^, which is controlled by the *L*.

**Figure 2:**
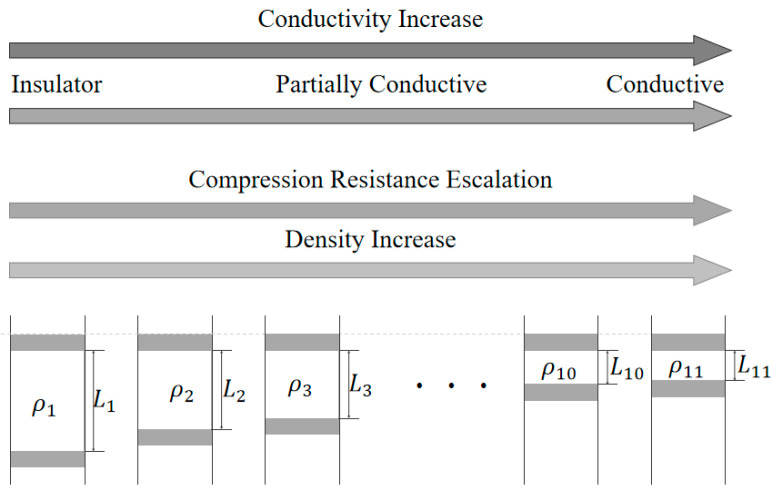
The performance of the CNT powder when pressing.

A jump in material density is caused by the press method. When the density of the CNT powder is small, it is easy to press, and the small increment causes small changes in density. When the density of the CNT powder is large, it is hard to press, and the small increment causes a large change. As shown in Figure 2, the conductivity of the CNT powder increases when the thickness *L* decreases. While the performance changes from insulator to conductor, the compression resistance escalates, and the density increases.

### 2.2. Parameters Measurement

**Figure 3:**
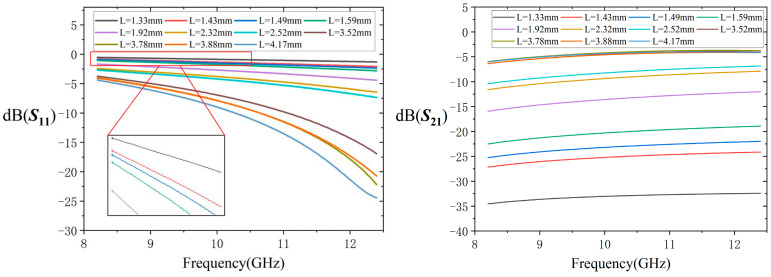
The measured S11 and S21 for the CNT powder.

The complex scattering parameters S_11_ and S_21_ of the waveguide filled with the sample were measured with a calibrated vector network analyzer (VNA) (model ZVA 40). The S21 of the waveguide is shown in Figure 3. Since the S21 is higher than −40 dB, which is the threshold for the transmission/reflection method [13], the Nicolson–Ross–Weir method can be used. The first step of de-embedding allows for determining the sample scattering parameters, which consider the electrical distances, L_01_ and L_0_, and the thickness of the CNTs, L. The A-parameters of the PVC in Figure 1b are computed as [14].

(2) $$\varepsilon_{1r},\mu_{1r},L_{01}\to [A^{PVC}]$$

The A-parameters of the air-filled section in Figure 1b are computed as

(3) $$\varepsilon_{0},\mu_{0},L_{0},L_{01},L\to [A^{Air}]$$

The experimentally measured S-parameters of the waveguide are then converted into A-parameters, namely,

(4) $$\left[S_{wav}^{\exp}]\to [A_{wav}^{\exp}\right]$$

Then, the A-parameters of the CNTs are expressed as

(5) $$[A^{CNTs}]={\left[A^{PVC}\right]}^{-1}[A_{wav}^{\exp}]{\left[A^{PVC}\right]}^{-1}{\left[A^{Air}\right]}^{-1}$$

The experimental A-parameters of the CNTs can subsequently be converted into S-parameters of the CNTs [15].

(6) $$\left[A^{CNTs}]\to [S^{CNTs}\right]$$

For the S-matrix of the samples, the standard Nicolson–Ross–Weir inversion method [16] was used to extract the complex permittivity (ε_r_) of the carbon nanotubes and the complex permeability (μ_r_), and the relevant closed-form equations are exhaustively given in the literature and will not be repeated here.

## 3. Results

**Figure 4:**
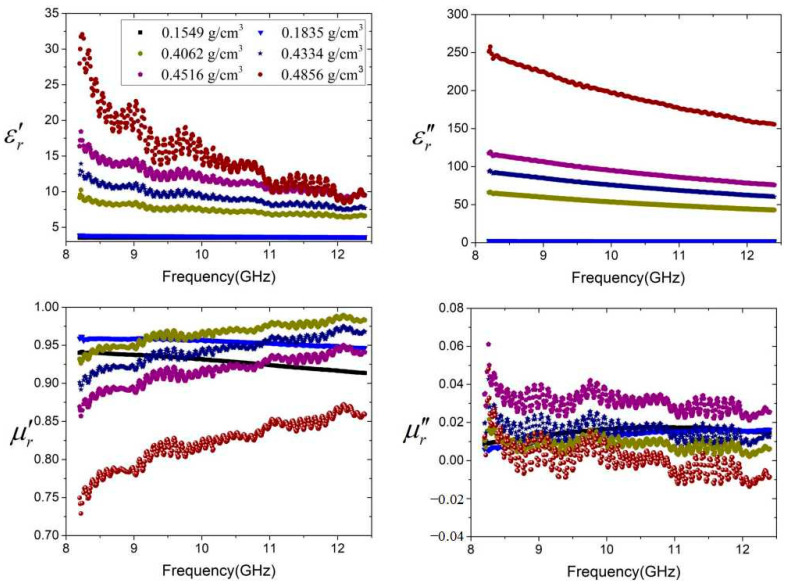
Variation in the extracted relative complex permittivity and permeability of the CNTs in powder form versus frequency at different packing densities.

The determined complex permittivity and permeability of the CNTs in the X-band for different packing densities are shown in Figure 4. It is evident that describing a clearly conductive material using dielectric parameters seems strange, although there is precedent for doing so. However, the permittivity can be transferred to conductivity in the microwave region [17].

(7) $$\begin{array}{l} \tan\delta =\frac{{\varepsilon_{r}}^{\prime \prime }}{{\varepsilon_{r}}^{\prime }}=\tan\delta_{d}+\frac{\sigma }{2\pi f\varepsilon_{0}{\varepsilon_{r}}^{\prime }}\\ \sigma =2\pi f\varepsilon_{0}\cdot \left({\varepsilon_{r}}^{\prime \prime }-{\varepsilon_{r}}^{\prime }\tan\delta_{d}\right) \end{array}$$

where tanδ represents the loss tangent angle, ${\varepsilon_{r}}^{\prime }$ is the real part of the complex permittivity, ${\varepsilon_{r}}^{\prime \prime }$ is the imaginary part of the complex permittivity, $\tan\delta_{d}$ is the dielectric losses, and σ denotes the conductivity. This formula illustrates the relationship between permittivity and conductivity in the microwave region. Additionally, the imaginary part of the complex permittivity exhibits frequency-dependent characteristics.

We observe quite a constant value of the determined parameters as a function of frequency when the packing density of CNTs is low, as it is dominated by air. The reason for this phenomenon is that the powder of CNTs is not connected with each other, and the electromagnetic waves just go through the sparse CNT powder shim.

Above a packing density of 0.4062 g/cm^3^, we observe a percolation behavior of permittivity versus frequency of CNTs, as shown in Figure 4, due to the dramatic increase in the number of nanotubes and the reduction in air interstices. As the density increases, the real part and the imaginary part of the complex permittivity increase. And the CNTs exhibited a dispersion phenomenon in the X-band. The values of the complex permittivity change with the frequencies. That is important for the design of the CNT interconnect line in the circuits.

The packing density has little effect on the complex permeability because the CNTs are nonmagnetic or low-magnetic. The graphs given in Figure 4 indicate that the highest value of the real part of relative complex permittivity of the CNTs, with the highest density obtained at 8.2 GHz, is 32.5, which then decreases continuously to finally reach 9.5 at 12.4 GHz. Similarly, the highest value of the imaginary part of the relative complex permittivity of the CNTs, with the highest density obtained at 8.2 GHz, is 250, and it gradually decreases to finally reach 160 at 12.4 GHz. Since the imaginary part of the permeability is close to 0, the measurement error may lead to a negative performance of the imaginary part [5]. The CNT in powder form changes from an insulator to a conductor and then becomes a good conductor. Then, the imaginary part of the permittivity increases more than 50 times. The black square is an illustration of 0.1549 g/cm^3^. The results are caused by the smoothness of the permittivity and permeability since the error of the measurement is small in this density.

To validate the proposed method, CST was used for simulation, and the simulation model was completely consistent with the actual measurement model. The materials under test are similar to the CNT powder with high density. It is important to note that the material properties in the simulations are idealized constants and do not represent the actual CNT behavior. Instead, they are benchmarks for validating the correctness of the proposed extraction method under controlled conditions. The relative complex permittivity of the material at the center frequency point was set to be 10-j50, the relative complex permeability is set to be 1-j0.02. The relative complex permittivity of the sheet for press is set to be 2-j0.01, the relative complex permeability of the sheet for press is set to be 1. The calculated results agree well with the true values. This kind of method is usually used for the validation [18,19].

## 4. Discussion

**Figure 5:**
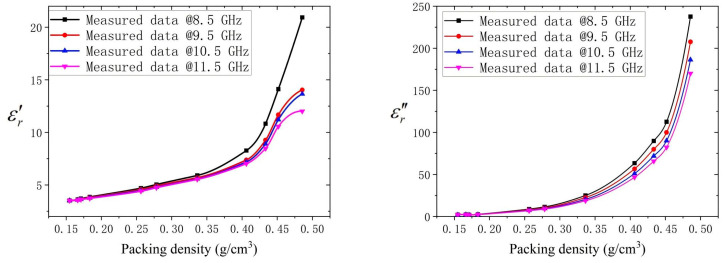
Variations in the ${\varepsilon_{r}}^{\prime }$ and the ${\varepsilon_{r}}^{\prime \prime }$ of the CNTs at 8.5, 9.5, 10.5, and 11.5 GHz.

The data in Figure 5 are obtained from the results shown in Figure 4 at 8.5, 9.5, 10.5, and 11.5 GHz, as well as other measurements with different packing densities. The experimental results show an enhancement of the complex permittivity with packing density. As the density increases, the ${\varepsilon_{r}}^{\prime }$ ranges from about 2.7 to more than 20, and the ${\varepsilon_{r}}^{\prime \prime }$ ranges from about 0 to 225 at 8.5 GHz. As the frequency increases, the range of variation decreases. That is, the dispersion characteristic decreases as the frequency increases.

## 5. Conclusions

Compared to the results in the literature, this study is the first to simultaneously determine the complex permittivity and permeability of CNTs in powder form with different densities. The results and the method are shown first. The frequency dependence of the determined parameters and the effects of the packing density of the CNTs are presented. The method could be intended for waveguides of other frequencies and coaxial line measurements. The method is suitable for any materials in powder form.

## Figures and Tables

**Table 1:** Changing the packing density of the CNTs by pressing the PVC.

*L* (mm)	4.17	3.88	3.78	3.52	2.52	2.32	1.92	1.59	1.49	1.43	1.33
*ρ* (g/cm^3^)	0.1549	0.1665	0.1709	0.1835	0.2563	0.2784	0.3364	0.4062	0.4334	0.4516	0.4856

## Data Availability

The original contributions presented in the study are included in the article, further inquiries can be directed to the corresponding author.
